# Antimicrobials in acute and long-term care: a point in time along the way to improved use

**DOI:** 10.2807/1560-7917.ES.2018.23.46.1800607

**Published:** 2018-11-15

**Authors:** Melinda M. Neuhauser, J. Todd Weber

**Affiliations:** 1 Division of Healthcare Quality Promotion, National Center for Emerging and Zoonotic Infectious Diseases, Centers for Disease Control and Prevention, Atlanta, United States

**Keywords:** antibiotic resistance, antimicrobial resistance, AMR, healthcare-associated infections, surveillance, antibiotic use, acute care, long-term care facilities, LTCF, point prevalence survey, PPS

Antimicrobial use is the most important modifiable factor contributing to resistance [1]. One key strategy against antimicrobial resistance that has the potential to improve patient outcomes is to optimise antimicrobial use. Understanding how antimicrobials are being used informs stewardship efforts in acute care, long-term care and outpatient settings [2]. In the acute care setting, stewardship programs encompass tracking and reporting aggregate antimicrobial use metrics, such as days of therapy or defined daily doses. Benchmarking use within and across facilities is helpful in identifying where action is needed. Antimicrobial use point prevalence surveys (PPS) complement the aggregate metrics by providing information on patient-level use, such as indication and site of infection during the specified time period [3,4]. This approach is able to reveal more targeted quality improvements and enables comparisons of antimicrobial use at the national, regional or local level. PPS may be particularly useful for resource-limited hospitals and long-term care facilities (LTCF) with restricted capabilities for capturing use data on a continual basis [5,6]. Since PPS evaluate antimicrobial use during a single time period, they need to be repeated at regular intervals to monitor trends over time.

On the occasion of the European Antibiotic Awareness Day on 18 November, this issue of *Eurosurveillance* is dedicated to several studies presenting results from European PPS based on the European Centre for Disease Prevention and Control (ECDC) healthcare-associated infections (HAI) and antimicrobial use protocol for acute care hospitals and the HAI and antimicrobial use protocol for LTCF [7,8].

Plachouras et al. describe the outcomes from the second European Union/European Economic Area (EU/EEA)-wide PPS conducted in acute care hospitals [3]. In this survey, the weighted prevalence of antimicrobial use was 30.5% (95% confidence interval (CI): 29.2–31.9%) in 1,209 acute care hospitals in 28 EU/EEA countries [3].

Since 2009, as part of a Transatlantic Taskforce on Antimicrobial Resistance, ECDC and the United States (US) Centers for Disease Control and Prevention (CDC) have collaborated to share and, where possible, harmonise methodologies for conducting PPS focused on HAI and antimicrobial use [9]. The European hospital-based antimicrobial use PPS coordinated by ECDC took place in 2011–12 and 2016–17; the CDC-led ones in the US were conducted in 2011 and 2015. In an analysis of a subset of the data from the US CDC’s hospital-based PPS conducted in 2015, a higher proportion of patients (non-weighted prevalence: 50.1%; 4,590/9,169) received at least one antimicrobial [10], compared to those in European hospitals (non-weighted prevalence: 32.9%; 102,093/310,755) [3]. Variation in methodologies between Europe and the US, including the definition of the prevalence time period (1 day vs 2 days) and data collectors, may have influenced these results. However, there was large variability in the point prevalence of antimicrobial use across European countries. Greece had the highest percentage at 55.6%, while Hungary had the lowest at 15.9% [3]. In the 2011 US PPS, variability by geographic region was not described [11]. The US also had a higher percentage of patients (50%) receiving two or more drugs [11], compared with most recent findings from European countries (30%) [3]. Overall, the antimicrobial use prevalence was similar between the first and second PPS in Europe, as well as when the US compared their results to the first and most recent US PPS survey. Both the European and US PPS revealed declining fluoroquinolone use, however, when compared with their first surveys [3,10].

In another study in this issue of *Eurosurveillance*, Karki et al. present results from the third point prevalence survey of HAI and antimicrobial use in European LTCF. The observed prevalence of antimicrobial use was 4.9% (95% CI: 4.8–5.1%) in 1,788 LTCF in 23 EU/EAA countries [4].

In the long-term care setting, the first US large-scale antimicrobial use PPS in 2017 comprised 15,295 residents in 161 nursing homes [12]. The results showed that 8.2% (95% CI: 7.8–8.7%) of residents received at least one antimicrobial at the time of the survey [12], compared with 4.9% (95% CI: 4.8–5.1%) of residents in the European prevalence survey [4]. In general, the European and US survey methodologies were more similar for the PPS in LTCF compared with that in acute care hospitals. The target population had notable differences; in the US, only nursing homes were surveyed, while in Europe nursing homes, residential homes and mixed LTCF were surveyed. Similar to acute care hospitals, prevalence in European LTCF varied geographically, with the highest values in Spain and Denmark (10.5%) and the lowest in Lithuania (0.7%) [4]. The urinary tract was the most common infection site listed as the source in both the US and European LTCF [4,12].

Descriptive antimicrobial use data from PPS are informative to guide stewardship efforts, but have limitations in addressing quality of prescribing for more targeted interventions. In order to address quality of prescribing, the CDC expanded data collection for the acute care PPS conducted in 2015 to describe the quality of antimicrobial drug prescribing in selected clinical circumstances, i.e. community-acquired pneumonia and urinary tract infection, and vancomycin and fluoroquinolone use [13]. The CDC, with input from external experts, is working to refine prescribing quality assessment pathways to describe opportunities for improvement in hospital prescribing practices.

The European Commission and the CDC have released recommendations regarding the key elements of antimicrobial stewardship programs in the acute care, long-term care and outpatient settings [14-17]. European Commission recommendations also target other key stakeholders such as local governments, prescribers, researchers and the pharmaceutical industry. The European healthcare-associated infections and antimicrobial use PPS included structure and process indicators for antimicrobial stewardship [3,4]. In acute care hospitals, approximately half of the hospitals have less than 0.1 full-time equivalent antimicrobial stewardship consultants per 250 beds, and approximately half of the European hospitals had a formal procedure for post-prescription review [3]. In the European LTCF survey, 39.4% of facilities had guidelines for appropriate antimicrobial use, 24.0% had a restrictive list of antimicrobials and 20.7% had annual training on appropriate antimicrobial prescribing [4]. In comparison, 59% of US nursing homes had guidelines for appropriate use, 25% had a restrictive list of antimicrobials and 73% had training for nursing staff (but ‘annual’ frequency was not specified in the questionnaire) [18]. Dedicating necessary resources in both acute care and LTCF settings is important to advance antimicrobial stewardship interventions.

In the US, stewardship programs in hospitals often target optimising antimicrobial therapy for commonly encountered infections such as community-acquired pneumonia, UTI, and skin and soft tissue infections [15]. Studies have demonstrated a number of interventions to improve antimicrobial use for each of these, making them likely high-yield targets for improvement.

Several important findings from the studies published in this issue can guide targeted stewardship program efforts. In the acute care PPS, surgical prophylaxis exceeded more than one day in 54.2% of the courses [3]. As noted by the authors, one preoperative dose is recommended for most surgical procedures, so optimising duration of therapy for surgical prophylaxis represents a stewardship opportunity to reduce unnecessary antimicrobial use, development of resistance and costs [3]. Further, documented indications for antimicrobials were frequently (19.8%) missing in the medical chart, which can be a barrier to improving use. The LTCF survey results showed that almost half (46.1%) of antimicrobials were prescribed for the urinary tract and the majority (74.0%) of antimicrobials were prescribed for prophylaxis of UTI [4]. Although quality of prescribing was not evaluated, optimising antimicrobial therapy for UTI represents another stewardship opportunity to reduce unnecessary prescribing for asymptomatic bacteriuria or medical prophylaxis.

The European PPS have contributed to our knowledge by highlighting that ca 30% and 5% of patients received at least one antimicrobial in acute care hospitals [3] and LTCF [4], respectively. Antibiotic use PPS provide a standardised methodology and data collection tool for facilities to extract and analyse data. These data can be used at the national, regional or local level to guide stewardship interventions. Examples for improving surgical prophylaxis duration in the hospital setting may include implementing standardised surgical prophylaxis protocols in collaboration with surgery and key stakeholders [19]. Often, more detailed quality assessment through a medication use evaluation (i.e. retrospective evaluation of clinical course for quality improvement) may be warranted to further identify more targeted interventions for commonly used antimicrobials or infections.

Identifying opportunities to streamline data collection is necessary, as PPS are currently performed by labour-intensive manual chart abstraction. As electronic health records continue to advance, leveraging electronic means to capture prevalence of HAI, antimicrobial use and quality of prescribing should be an aspiration. For example, the US Department of Veterans Affairs Salt Lake City IDEAS Center has begun to capture electronic medication use evaluations for community-acquired pneumonia [20] and other common clinical conditions.

Although PPS are complicated and time-consuming efforts, they are likely more feasible for resource-limited hospitals and LTCF than creating a prospective antimicrobial use surveillance system. With many countries around the world performing antimicrobial use PPS, there is an opportunity for global collaboration in order to share information and knowledge towards the goal of more judicious use of precious, lifesaving antimicrobials.
